# A15 *GIARDIA DUODENALIS* INDUCES REGIOSPECIFIC ALTERATIONS TO INTESTINAL MUCOSAL GLYCOSYLATION PATTERNS

**DOI:** 10.1093/jcag/gwab049.014

**Published:** 2022-02-21

**Authors:** E Fekete, T Allain, A Buret

**Affiliations:** 1 Biological Sciences, University of Calgary, Calgary, AB, Canada; 2 University of Calgary, Calgary, AB, Canada

## Abstract

**Background:**

Disruption of intestinal barrier function is important in the pathogenesis of numerous intestinal diseases and can lead to increased intestinal permeability. During infection with the protozoan parasite *Giardia duodenalis*, intestinal permeability is increased in part due to disruption of the intestinal mucus barrier. Intestinal mucus is composed primarily of the heavily glycosylated MUC2 mucin. Glycosylation of mucins is important for maintaining the structure of the mucus gel, and glycans are key mediators of host-microbe interactions, as they provide binding sites to microbes and can be degraded as a nutrient source. Changes to mucin glycosylation patterns have been noted during intestinal inflammation and bacterial infection, and may contribute to altered intestinal permeability and dysbiosis. We hypothesized that intestinal mucosal glycosylation patterns may be disrupted during *Giardia* infection, and that this may contribute to *Giardia*-induced barrier dysfunction.

**Aims:**

Characterize changes to mucosal glycosylation patterns in the small and large intestines during *in vivo Giardia* infection.

**Methods:**

3–4 week old C57BL/6 mice were infected with *Giardia duodenalis* strain GS/M for 7 days. Tissue sections from the jejunum and colon were collected and stained with various fluorescein-coupled lectins (CONA, DBA, PNA, WGA, SNA, UEA-1) and fluorescence was quantified and normalized to tissue area. Quantitative PCR (qPCR) was performed for glycosyltransferase genes in the jejunum and colon.

**Results:**

In the jejunum, abundance of N-acetylglucosamine increased upon infection, while sialic acid and fucose abundance were significantly decreased in infected mice compared to controls. Conversely, in the distal colon, mannose and sialic acid abundance increased significantly upon infection.

Expression of mucin-associated glycosyltransferase genes was also altered in the small and large intestines of *Giardia* infected mice. In the jejunums of infected mice, expression of the sulfotransferase Chst4 decreased, while the fucosyltransferase Fut2 and the sialyltransferase St6GalNAc1 increased in comparison to controls. In both the jejunum and distal colon, expression of the core 2 synthase C2GnT1 increased, while expression of the core 1 synthase C1GalT1 was similar between control and infected mice.

**Conclusions:**

Glycosylation patterns and the expression of glycosyltransferase genes are altered in both the small and large intestines of *Giardia*-infected mice. Disruptions to mucin glycans appear to be regiospecific, suggesting that unique mechanisms are involved in the regulation of mucosal glycosylation during parasitic infection throughout different regions of the gut. These findings uncover a novel mechanism in the pathogenesis of *Giardia* infection, and may be important in understanding intestinal barrier dysfunction in a variety of different gastrointestinal diseases.

**Funding Agencies:**

CCC

